# Treatment of Staggers

**Published:** 1894-01

**Authors:** M. Parazols


### TREATMENT OF STAGGERS.

By M. Parazols.

M. Racca, Military Veterinarian, has already reported two cases of staggers cured by hypodermic injections of pilocarpine. M. Parazols has tried the treatment which gave such good returns to his Italian colleague and congratulates himself on the result. He reports three cases where horses affected with staggers were cured in less than a week by injections of the nitrate and chloro-hydrate of pilocarpine. The dose was 60 cgm. the first day, 78 cgm. the second and 80 cgm. the third. The three injections were sufficient. The author does not claim that pilocarpine will cure all cases; but when the immobility is due to effusion into the lateral ventricles it will surely give good results, whilst it certainly will not when due to a tumor.—*Revue Vétérinaire.*

